# Maternal but not fetoplacental health can be improved by metformin in a murine diet-induced model of maternal obesity and glucose intolerance

**DOI:** 10.1113/JP281902

**Published:** 2021-09-29

**Authors:** Antonia Hufnagel, Denise S Fernandez-Twinn, Heather L Blackmore, Thomas J Ashmore, Robert A Heaton, Benjamin Jenkins, Albert Koulman, Iain P Hargreaves, Catherine E Aiken, Susan E Ozanne

**Affiliations:** 1 University of Cambridge Metabolic Research Laboratories and MRC Metabolic Diseases Unit, Wellcome Trust-MRC Institute of Metabolic Science, Level 4, Addenbrooke’s Hospital, Cambridge, Cambridgeshire, United Kingdom, CB22 0QQ; 2 School of Pharmacy and Biomolecular Sciences, Liverpool John Moores University, Liverpool L3 3AF, UK; 3 Department of Obstetrics and Gynaecology, University of Cambridge, Cambridge, United Kingdom; National Institute for Health Research Cambridge Biomedical Research Centre, Cambridge, University of Cambridge, United Kingdom

**Keywords:** developmental programming, gestational diabetes mellitus, maternal obesity, metformin, placenta

## Abstract

Maternal obesity is a global problem that increases the risk of short- and long-term adverse outcomes for mother and child, many of which are linked to gestational diabetes mellitus. Effective treatments are essential to prevent the transmission of poor metabolic health from mother to child. Metformin is an effective glucose lowering drug commonly used to treat gestational diabetes mellitus; however, its wider effects on maternal and fetal health are poorly explored. In this study we used a mouse (C57Bl6/J) model of diet-induced (high sugar/high fat) maternal obesity to explore the impact of metformin on maternal and feto-placental health. Metformin (300 mg/kg/day) was given to obese females via the diet and was shown to achieve clinically relevant concentrations in maternal serum (1669±568 nM in late pregnancy). Obese dams developed glucose intolerance during pregnancy and had reduced uterine artery compliance. Metformin treatment of obese dams improved maternal glucose tolerance, reduced maternal fat mass, and restored uterine artery function. Placental efficiency was reduced in obese dams, with increased calcification and reduced labyrinthine area. Consequently, fetuses from obese dams weighed less (*p*<0.001) at the end of gestation. Despite normalisation of maternal parameters, metformin did not correct placental structure or fetal growth restriction. Metformin levels were substantial in the placenta and fetal circulation (109.7±125.4 nmol/g in the placenta and 2063±2327 nM in fetal plasma). These findings reveal the distinct effects of metformin administration during pregnancy on mother and fetus and highlight the complex balance of risk versus benefits that are weighed in obstetric medical treatments.

## Graphical abstract

**Abstract Figure:**
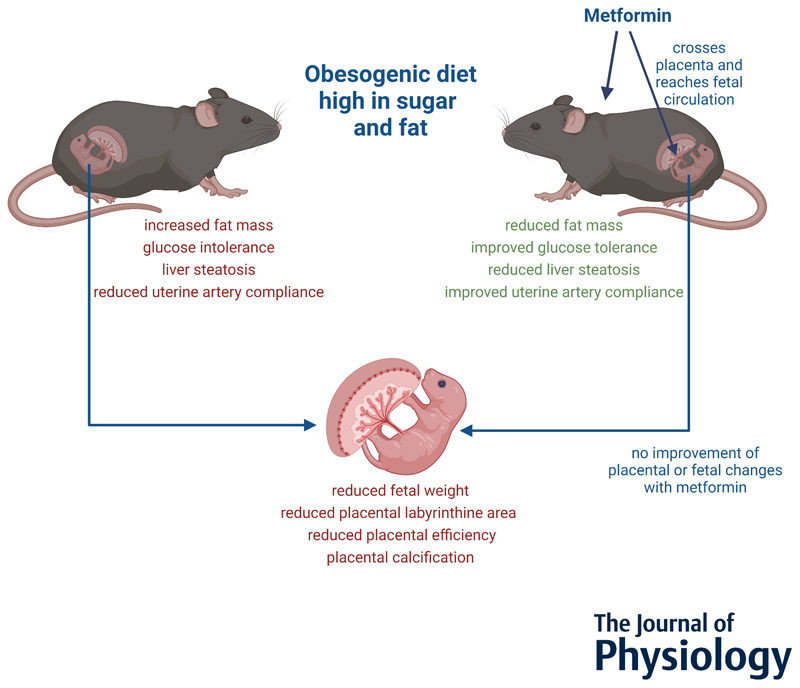
Obesogenic diet during pregnancy leads to impaired metabolic health in the mother and reduced fetal growth and placental pathologies. Maternal metabolic health can be markedly improved by metformin treatment, however this is not the case for fetal and placental health and metformin reaches the placenta and fetal circulation which could have direct short- and long-term effects.

## Introduction

The growing prevalence of obesity worldwide means that in many populations at least 50% of women are overweight or obese at the start of pregnancy (Hill *et al.,* 2019). Obesity during pregnancy is associated with increased risk of complications, including preeclampsia, preterm delivery, stillbirth, and importantly gestational diabetes mellitus (GDM) (Stephenson *et al.,* 2018). It is now estimated that the prevalence of GDM ranges from 1% to 30% worldwide (Mcintyre *et al.,* 2019).

Maternal obesity and untreated GDM during pregnancy have direct effects on the fetus, with implications for long-term offspring health (Alfaradhi & Ozanne, 2011). Observational studies in humans show increased risks of obesity (Hu *et al.,* 2019), type 2 diabetes (Lahti-Pulkkinen *et al.,* 2019), and cardiovascular disease (Gaillard, 2015) in offspring born to obese mothers and those with GDM (Mitanchez *et al.,* 2015). Studies in animal models by our laboratory and others have shown previously that these relationships are causal. These studies demonstrate that obesity and/or glucose intolerance during pregnancy lead to cardiac dysfunction (Blackmore *et al.,* 2014), insulin resistance (Isganaitis *et al.,* 2014), hyperphagia (Steculorum & Bouret, 2011), obesity (Samuelsson *et al.,* 2008) and fatty liver (Alfaradhi *et al.,* 2014) in young adult offspring. However, the mechanisms linking fetal development and growth in affected pregnancies with long-term adverse effects are complex and yet to be fully understood.

The placenta is the key interface between the mother and fetus, and therefore a likely mediator of the effects of maternal health on the developing fetus. Studies in humans and in animal models have shown that placentas from obese pregnancies display lipotoxicity (Jarvie *et al.,* 2010), inflammation (Pantham *et al.,* 2015), and have reduced placental vessel density (Hayes *et al.,* 2012), highlighting that the protective capacities of the placenta can be exhausted in diabetic and/or obese pregnancies (Desoye & Wells, 2021).

Interventions need to be carefully assessed to improve maternal and fetal health. Lifestyle and dietary interventions are generally the first recommendation to treat GDM, and are successful in >50% of women (ADA, 2019). If these interventions fail, pharmacological interventions such as metformin, glyburide, or insulin are implemented (SMFM, 2018). Metformin, a biguanide with glucose-lowering actions, is a pragmatic alternative to insulin as it can be taken orally, does not need to be refrigerated, and does not cause hypoglycaemic episodes (Gray *et al.,* 2017). In the UK, National Institute for Health and Care Excellence (NICE) guidelines recommend metformin as a first-line drug therapy for GDM (NICE, 2015) whereas other countries, such as Germany and Turkey (Schäfer-Graf *et al.,* 2018; SEMT, 2019), are much more cautious regarding metformin use in pregnancy.

It is well-established that metformin treatment of GDM improves glycaemic control in the mother and is associated with reduced gestational weight gain (Syngelaki *et al.,* 2016). However, there is relatively little data regarding immediate or long-term effects of maternal metformin use on the offspring (Tarry-Adkins *et al.,* 2019). Unlike insulin, metformin freely crosses the placenta and reaches circulating concentrations in the fetus that match those in the mother (Priya & Kalra, 2018). Human studies looking at polycystic ovary syndrome, GDM, and type 2 diabetes pregnancies suggest that intrauterine metformin exposure leads to reduced birthweight followed by increased adiposity later in childhood (Rowan *et al.,* 2011; Guro *et al.,* 2018; Feig *et al.,* 2020). However data on immediate effects of metformin on the fetus and placental function are scarce (Tarry-Adkins *et al.,* 2019) despite the possibility that metformin could have potential negative effects on the placenta and fetal development due to its inhibition of the mTOR pathway, cell proliferation and mitochondrial function (Lindsay & Loeken, 2017).

We addressed this knowledge gap by characterising maternal metabolic health, fetal growth, and placental structure and function using a murine model of metformin treatment for diet-induced obesity and glucose intolerance in pregnancy.

## Methods

### Ethical approval

Animal studies were carried out following review and approval by the University of Cambridge Animal Welfare Ethical Review Body and in accordance with the UK Animals Scientific Procedures Act 1986. The study was performed under the animal project licence P5FDF0206 issued by the UK Home Office and complies with the standards stated for the Journal of Physiology (Grundy, 2015).

### Animal work

A model of maternal diet-induced obesity that is well-established in our laboratory and is described in detail elsewhere (Fernandez-Twinn *et al.,* 2012) was used. Mice were purchased from Charles River Laboratories (Cat#000664, RRID: IMSR_JAX:000664) and bred in house. For all measurements [other than intraperitoneal glucose tolerance tests (ipGTT)] 14 control, 14 obese and 13 obese metformin-treated animals were included. For ipGTT, power calculations demonstrated a greater n was required, hence 20 control, 20 obese and 19 obese metformin-treated animals were used for this measurement.

After weaning at 3 weeks of age, female C57Bl6/J mice were fed *ad libitum* either an obesogenic diet high in sugar and fat (10% simple sugars, 20% animal fat, 23% protein [w/w], 4.5 kcal/g, Special Dietary Services, Cat #824053) together with condensed milk in glass pots (55% simple sugar, 8% fat, 8% protein [w/w], 3.2 kcal/g, Nestle, Cat #12029969) and a mineral mix (MP Biomedicals, Cat #AIN93G) or a control chow diet (RM1, 7% simple sugars, 3% fat, 15% protein [w/w], 3.5 kcal/g, Special Dietary Services, Cat #801002). In this model there is no difference in protein intake between the groups as shown previously (Samuelsson *et al.,* 2008; Maragkoudaki *et al.,* 2020). Assignment of dietary groups was carried out by an animal technician who was not involved in any of the subsequent physiological or molecular analyses. Mice were then mated for a first pregnancy at 6 weeks of age after which they were allowed at least one week of rest for recovery. Animals on a control diet were mated for the second experimental pregnancy with a body weight ≤25 g. Mice fed an obesogenic diet were mated or dosed with metformin once they reached a body weight of ≥35 g. For all groups this was at approximately 18 weeks of age. Mice were single-housed and kept in individually ventilated cages with wood chip bedding, free access to food, water, and environmental enrichment (nesting material and a tunnel) in a 12 h light/dark cycle. Metformin (MP Biomedicals Cat #02151691-CF) was administered one week prior to mating and throughout pregnancy in the condensed milk. Weighing of condensed milk twice a week allowed the calculation and adjustment of metformin intake in mg/kg/day. The average dose that animals received was 255.2 ± 48.0 mg/kg/day, which lies in the desired range of 200 - 300 mg/kg/day, based on clinically relevant doses (Salomäki *et al.,* 2013). Liquid chromatography - mass spectrometry (LC-MS) showed the mean serum metformin concentration was 1669 ± 568 nM, which falls within the clinical range reported in human pregnancies (Liao *et al.,* 2020). The study was designed to address whether intervention with metformin can improve detrimental effects of a pregnancy complicated by obesity and GDM, therefore no metformin-treated control group was used. As metformin is not given to lean pregnant women this is not clinically relevant. This is in line with the ARRIVE guidelines (NC3Rs) so that the minimal number of animals needed is used.

### Intraperitoneal GTT

Dams were fasted for 4 hours on the morning of embryonic day E17.5. Glucose (1mg/kg) was injected intraperitoneally, and blood glucose levels measured at 0, 15, 30, 60 and 120 minutes using a glucometer (AlphaTRAK, Abbot Logistics). Collection of tail blood was performed at 0 minutes into capillary tubes (Hirschmann-Laborgerate). Glucose curves are shown as a percentage of starting glucose. If the glucose levels rose less than 50% between fasting and timepoint 15 and or 30 minutes the GTT data was excluded. Insulin was measured with the Crystal Chem Mouse Insulin ELISA (Ultra-Sensitive) kit (Cat #90080). The HOMA-IR was calculated according to the following formula: fasting insulin [mU/l] x fasting glucose [mmol/l]/22.5.

### Fat mass assessment

Fat mass at E18.5 was assessed via Time Domain Nuclear Magnetic Resonance (TD-NMR, Bruker) measurements.

### Ultrasound assessments

Uterine, umbilical and fetal middle cerebral artery function were assessed via ultrasound in the morning of day E18.5 (FUJIFILM VisualSonics, Vevo3100). Anaesthesia in the dams was induced with inhalation of 2% isoflurane and then maintained at 1.5% isoflurane. Isoflurane is commonly used in ultrasound as it affects heart and respiration rate the least compared to other anaesthetics (Janssen *et al.,* 2004). Mice were placed on a heated platform, the electrocardiogram monitored and body temperature measured via a rectal probe and kept at around 36°C throughout. Uterine artery Doppler measurements were obtained by using the bladder and the split of the uterine and iliac artery from the abdominal aorta as landmarks (Zhang & Croy, 2009). The pulsatility and resistance index of the uterine artery were corrected for maternal heart rate (Ochi *et al.,* 2003). Both indices are a surrogate measure for the vessel resistance and vascular compliance (Holmgren *et al.,* 2020). The fetal Middle Cerebral Artery (MCA) was scanned in a cross-sectional view of the head at the level of the circle of Willis and the umbilical artery was measured in a free loop transverse section (Hernandez-Andrade *et al.,* 2014). The cerebroplacental ratio (CPR) was calculated via dividing the pulsatility index of the MCA by that of the umbilical artery. Scanned fetuses were marked on the skin of the dam so that they could be identified and sexed upon dissection. The Placental Pulsatility Index (PPI) was calculated using the following formula: PPI = (uterine artery PI + umbilical artery PI)/2 (Gudmundsson *et al.,* 2017). Analysis of the ultrasound recordings was performed with the VevoLab software.

### Dissections

After the ultrasound measurements were taken, cardiac puncture was performed under 2% isoflurane anaesthesia and death confirmed by cervical dislocation. Tissues of the dams were collected, and fetuses and placentas dissected out. Fetuses were killed by immersion in cold PBS. Fetal weight was recorded, and biometry measured with a caliper. Amniotic fluid was taken from the intact amniotic sac via a syringe. Fetal blood was obtained by collecting the blood after decapitation into capillary tubes. Fetal liver and kidneys were dissected out, quickly frozen on dry ice and stored at -80°C. Fetuses were sexed visually by detection of a black spot between tail and genital tubercle present in male fetuses (Deeney *et al.,* 2016) and subsequently confirmed via molecular analysis based on a protocol from McFarlane et al. (Mcfarlane *et al.,* 2013). Briefly, genomic DNA was isolated, and PCR performed (GO Taq G2 DNA polymerase from Promega, Cat #PAM7841, annealing temperature 57°C) with the following primer: SX_F, 5ʹ-GATGATTTGAGTGGAAATGTGAGGTA-3ʹ; SX_R, 5ʹ-CTTATGTTTATAGGCATG CACCATGTA-3ʹ. On an agarose gel male samples display one band at 280bp, female samples show 2-3 bands (480bp, 660bp, 685bp).

### sFlt measurement

sFlt (VEGF-R1) was measured in maternal serum by ELISA according to the manufacturer’s instructions (R&D Systems, Cat #MVR100).

### Histology (liver and placenta)

Dam livers and placentas were immersion-fixed in 10% formalin and processed. Dam liver sections (one mid-section, 5μm) were stained with Haematoxylin and Eosin and fat vacuole content quantified with the HALO software (Indica labs). Artificial intelligence of the software was used to exclude vessels for the subsequent analysis of the fat vacuoles, via the HALO vacuole quantification module.

Placental sections (one mid-section, 5 μm) were deparaffinised and rehydrated and incubated in water for 15 mins. at 60°C. Antigen retrieval was performed (97°C, pH=9, 20 mins., Vector, Cat #H-3301) and the slides blocked with 1x animal-free blocking solution for 1 hour (Vector, Cat #SP-5030). Slides were incubated with the primary antibodies for CD31 and Tpbpa (R&D, Cat #AF3628, RRID: AB_2161028 1:40 dilution and abcam, Cat #ab104401, RRID: AB_10901888, 1:1000 dilution in antibody diluent (Vector, Cat #SP-5035)) overnight at 4°C. After washing (0.5% Tween in TBS, T-TBS) the secondary antibodies were applied subsequently for 1 hour at room temperature (first NL557 (R&D, Cat# NL001, RRID: AB_663766) at 1:200, then Alexa488 (Invitrogen, Cat# A11008, RRID: AB_143165) at 1:1000). After washes in T-TBS and PBS slides were stained with DAPI for 10 min. in the dark and TrueVIEW quenching solution (Vector, SP-4800) was subsequently used according to the protocol. Slides were mounted in Vectashield hard set anti-fade mounting medium (Vector, Cat #H-1400). Analysis of the slides was performed blinded with HALO software by manually delineating the placental layers.

To analyse placental calcification, Alizarin Red staining was performed (Orchard, 2013). Sections were dewaxed and immediately put into 95% alcohol, slides were air-dried and incubated in Alizarin Red (Sigma-Aldrich, Cat #A-5533) solution for 5 minutes (1% aqueous solution pH=6.4, ammonium hydroxide). After rinsing under water, slides were counterstained with fast green (0.05% FCF (Sigma-Aldrich, Cat #F-7252) in 0.2% acetic acid for 15 seconds). Slides were washed under water, dehydrated, cleared and mounted in synthetic resin. Slides were analysed automatically via the HALO software with a classifier programmed to count Alizarin Red positive and negative areas within the manually delineated whole placental section.

### Quantitative RT-PCR

RNA from placentas, fetal livers and kidneys (5 mg) was extracted with a miRNeasy Micro kit (Qiagen, Cat #217084), 1 fetus per sex and litter with an n of 3 was used. Fetal liver and kidney were chosen as two metabolic tissues that can be clearly dissected out in the E18.5 fetus. DNA was digested on column with a DNase Qiagen set (Qiagen, Cat #79254). RNA concentrations were measured with the Nanodrop and concentrations were between 300 and 700 ng/μl for all samples with a A260/280 ratio between 2 and 2.15. RNA was heated at 65°C prior to reverse transcription. Reverse transcription was performed with 1μg total RNA in a 20 μl reaction with the RevertAid kit (Thermo Scientific, Cat #K1691) using the following protocol: 5 mins. at 25°C, 60 mins. at 42°C and 5 mins at 70°C. QPCR was performed with the TaqMan Master Mix (Thermo Scientific, Cat #4304437) in a 7 μl reaction using a dilution of 1:16 of the RT reaction mix. All TaqMan probes were purchased from Thermo Scientific and are listed in table 1 below. The qPCR was performed on a QuantStudio 7 Flex Real-Time PCR system for 384 wells using the following cycling conditions: 2 mins. at 50°C, 10 mins at 95°C and then 15 secs. at 95°C and 1 min. at 60°C for 40 cycles. The MIQE guidelines were followed for the quantitative RT-PCR (Bustin et al., 2009).

### Liquid Chromatography - Mass Spectrometry (LC-MS)

Metformin and metformin-d6 were purchased from Sigma Aldrich (Cat #PHR1084) and QMX laboratories (Cat #D-6012) and all solvents/additives were at least HPLC grade. Metformin was extracted as previously described (Jenkins *et al.,* 2020). Briefly, the samples were weighed/pipetted into plastic tubes (Eppendorf) with a 5 mm stainless steel ball. Then, 400 μL of chloroform: methanol (2:1, Sigma Aldrich, Cat #34854 and Cat #M/4056/17) solution was added. The samples were then homogenised using a Bioprep-24-1004 homogeniser (Allsheng) run at 4.5 m/s for 60 seconds. 100 μL of the metformin-d6 (1 μM in water) was added followed by the addition of 600 μL of chloroform: methanol (2:1) solution and 300 μL of water (Sigma Aldrich, Cat #1.15333). The samples were vortexed and centrifuged at ~21,000 g for 5 minutes. The aqueous extracts were transferred into glass vials and dried down using a Concentrator Plus (Eppendorf) run at 60°C for 180 minutes. The samples were reconstituted in 100 μL of chromatography starting conditions and transferred into glass vial inserts for analysis. LC-MS analysis was achieved using a HPLC System (Shimadzu UK Limited) injecting 5 μL of the sample onto a Scherzo SM-C18 column (150 mm * 3 mm I.D., 3 μm) maintained at 40°C. Mobile phase [A] was 30 mM ammonium acetate (Sigma Aldrich, Cat #17836) in water with 0.02% acetic acid (Sigma Aldrich, Cat # 222142500). Mobile phase [B] was 20% acetonitrile (Sigma Aldrich, Cat #34851), 80% water with 0.8% acetic acid. The flow was maintained at 0.5 mL/min with the following gradient: 0.00 minutes_10% [B]; 0.20 min_10% [B]; 1.20 min_99% [B]; 5.00 min_99% [B]; 5.10 min_10% [B]; 8.00 min_10% [B]. The needle was washed using 50:50 water: acetonitrile solution. An Exactive Orbitrap with a heated electrospray ionisation source (Thermo Fisher Scientific) was calibrated before sample analysis. The instrument tune file (positive mode, full-scan: m/z 100 to 200, resolution: 2 Hz) was optimised for metformin and applied throughout the analysis.

### Statistical analyses

The visual difference in body weight of control and obese dams means that it is not possible to blind the individual carrying out the physiological analysis to maternal group. However histological analysis was performed following coding and blinding of the sample group to the individual carrying out the analysis. One- and Two-Way ANOVA, Pearson correlation and paired t-test were performed with the GraphPad Prism 9.0.0 Software, statistical outliers were removed following Rout testing and n numbers are indicated below each Figure and mean ± SD presented in the text and the Figures. Assumptions for the use of parametric tests were tested via the Shapiro-Wilk test for normality and the Brown-Forsythe test for equal variance. The fasting insulin, ipGTT AUC, HOMA-IR and liver steatosis data showed unequal variance and therefore a Welch ANOVA was performed. All other data met the assumptions required. The heatmap, organisation of the data, calculations and linear models were performed in R Studio (Version 1.3.959). Random-effects models were constructed for the fetal data (using the lmer4 package in R) to account for litter structure as a random effect, with sex and the experimental group as fixed effects. Other possible co-variates, for example litter size and position within the uterus, did not significantly improve the fit of the model and were therefore not included in the final model for analysis. The model was used for the analysis of the fetal bodyweight, fetal biometrical measurements, the placental weight and the body weight to placental ratio allowing all fetuses in a litter to be included in the analysis, however for visualisation the mean ± SD is presented. For the analysis of the fetal liver weight, the placental structure (placental labyrinth and Calcium), the umbilical artery pulsatility index, the PPI, the litter size, the MCA and CPR one fetus per litter and sex was analysed and analysis performed with a Two-Way ANOVA with maternal environment and fetal sex as the independent variables. Whenever a subset of these animals was used for a measure the n numbers are indicated in the Figure legend or in the text if a Figure is not shown.

## Results

### Equal concentrations of metformin are found in the maternal circulation and fetal circulation

**Figure 1:**
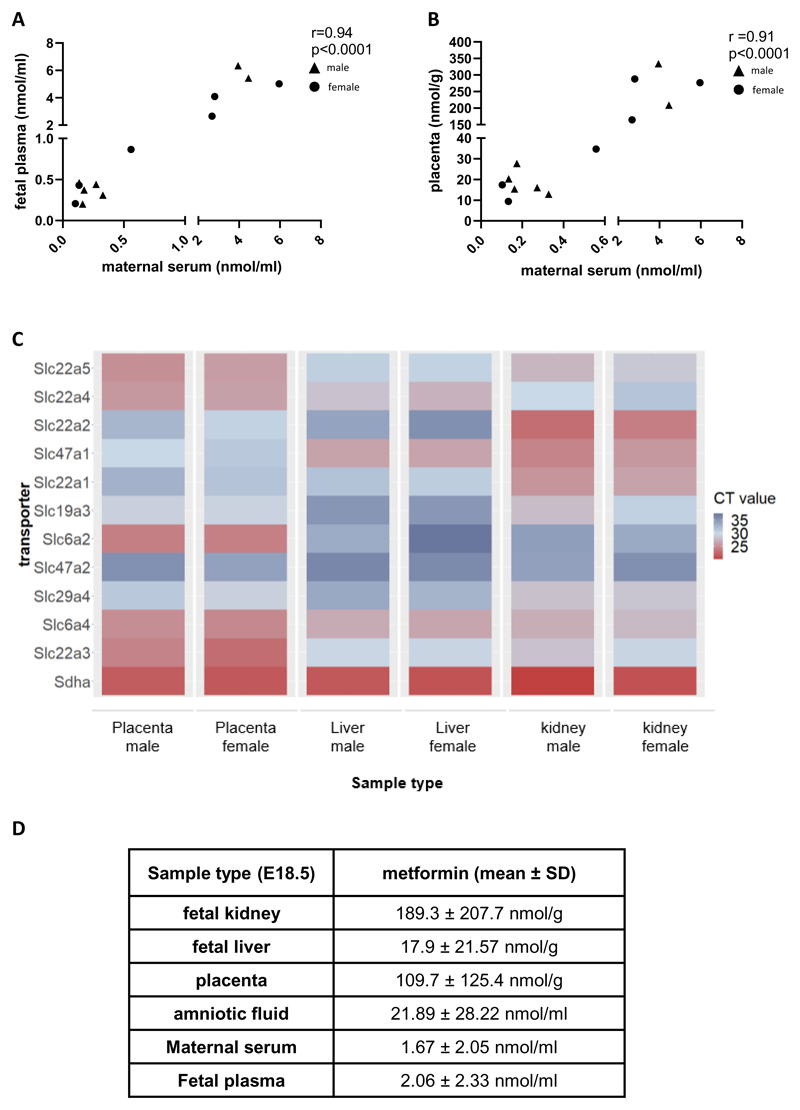
Metformin given to the obese mum during pregnancy can reach the fetal circulation and fetal tissues (A) Metformin concentrations measured in maternal serum were correlated with fetal plasma concentrations and (B) metformin levels in the placenta, circles represent female, triangles represent male fetuses. Linear regression and Pearson correlation coefficient r are shown. (C) Expression of eleven transporters (known for their ability to transport metformin) was analysed in E18.5 placenta, fetal liver and fetal kidney, n=3 per tissue type and sex respectively. Raw CT values are shown, ranging from high expression (low CT values, red color) to low expression (high CT values, blue color) and Sdha included as an endogenous control. (D) Metformin was measured in fetal kidney, liver, amniotic fluid (n=3 female and n=3 male) and placentas (n=7 male, n=6 female) via LC-MS, fluid samples are expressed in nmol/mL and tissue samples in nmol/g to allow an approximate comparison.

Metformin concentrations in maternal serum (1.67 ± 2.05 nmol/mL) on E18.5 of pregnancy were comparable to those previously reported in pregnant women being treated for GDM (Liao *et al.,* 2020). Furthermore, comparable concentrations were present in fetal plasma (2.06 ± 2.33 nmol/mL, n=13) and the placenta (109.7 ± 125.4 nmol/g, n=13) on E18.5 of pregnancy. There was therefore a strong positive correlation between maternal serum, fetal plasma, and placental metformin concentrations at this time point (Fig. 1*A, B*). Metformin was also detected at high levels in E13.5 placenta (143.8 ± 118.2 nmol/g, n=4). Consistent with the uptake of metformin into placental tissue, high levels of expression of 5 of the 11 known metformin transporters (Slc22a3, Slc22a4, Slc22a5, Slc6a2, and Slc6a4) were detected in the placenta at E18.5 (Fig. 1*C*). Of these Slc22a3 (Oct3), Slc6a2 (Net), Slc6a4 (Sert) were expressed at a higher level than Slc22a5 (Octn2) and Slc22a4 (Octn1) (Fig. 1*C*). Additional evidence for metformin passing into fetal circulation was demonstrated by its detection in fetal liver but also at significantly higher levels in fetal kidneys (Fig. 1*D*, p=0.02 for kidney *vs.* liver comparison via Mann-Whitney test). Accordingly, seven transporters were present in the fetal kidneys: Slc22a2 (Oct2), Slc22a3 (Oct3), Slc22a5 (Octn2), Slc6a4 (Sert), Slc47a1 (Mate1) and Slc22a1 (Oct1) (Fig 1*C*). In the liver only three transporters (Slc22a4 (Octn1), Slc47a1 (Mate1), Slc6a4 (Sert)) were detected (Fig. 1*C*). Consistent with highest observed levels of metformin and metformin transporters in the fetal kidneys, and consistent with urinary excretion, metformin was also detected in the amniotic fluid (Fig 1*D*).

### Metformin treatment in obese dams reduces fat mass at the end of pregnancy

**Figure 2:**
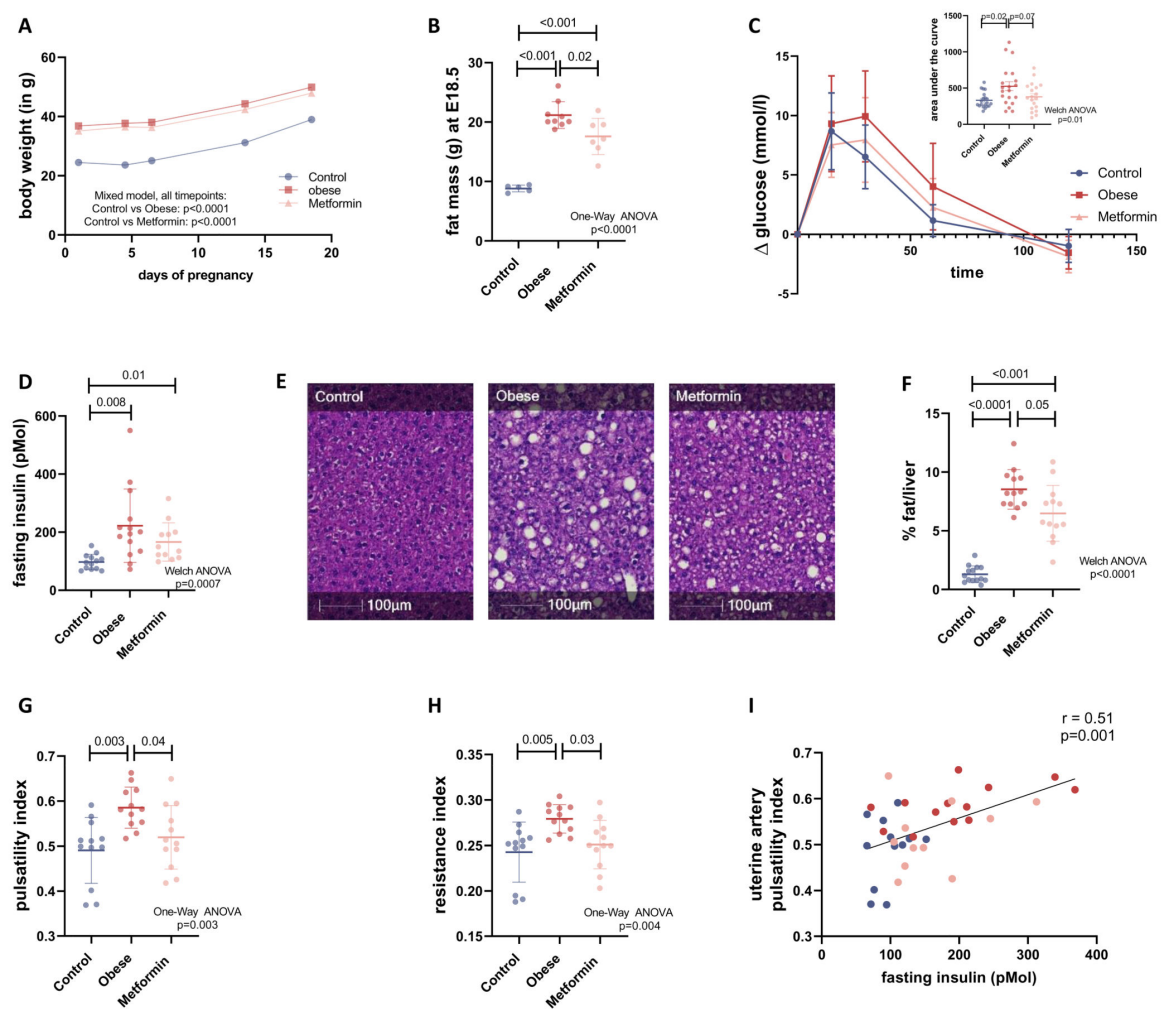
Maternal characteristics (A) Bodyweight of the dams was measured at day of the plug (E0.5), day 4.5, 6.5, 13.5 and 18.5 of gestation (n=14 for control, n=14 for obese, n=13 for metformin dams), mean ± SD and mixed model analysis is shown. (B) On day 18.5 fat mass was measured via TD-NMR (n=5 for control, n=9 for obese, n=7 for metformin dams). (C). An ipGTT was performed after a 4-hour fast on day 17.5 of pregnancy and glucose levels were measured and presented as the difference to the starting glucose level (n=20 for control, n=20 for obese, n=19 for metformin dams). The insert Figure shows the area under the curve, mean ± SD and Welch ANOVA analysis are shown. (D) Fasting insulin levels were measured and mean ± SD and Welch ANOVA analysis are shown (n=13 for control, n=14 for obese, n=13 for metformin dams). (E) Liver sections were stained with Haematoxylin and Eosin (representative images shown) and (F) the fat vacuole content quantified with HALO image analysis platform as a percentage of the whole liver section (n=14 for control, n=13 for obese, n=14 for metformin dams). (G) Pulsatility and (H) resistance indices were calculated and corrected for individual maternal heart rates (n=12 for all three groups). (I) Fasting insulin levels were correlated with uterine artery pulsatility index and linear correlation and Pearson correlation coefficient r are shown. If not indicated differently statistical analyses in the Figure show One-Way ANOVA followed by Tukey’s multiple comparison test, error bars show mean ± SD.

Dams randomised to the obesogenic diet were heavier throughout pregnancy compared to those randomised to control diet (Fig. 2A). At the end of gestation (E18.5), obese untreated dams had a significantly higher fat mass than the controls (*p*<0.0001, Fig. 2B). Supplementing the obese diet with metformin resulted in a significantly lower fat mass in the obese metformin-treated group compared to the obese untreated group at E18.5 (*p*=0.02, Fig. 2B).

### Metformin supplementation to obese dams improves glucose tolerance and liver steatosis

Glucose levels after a 4-hour fast were not significantly different between the three groups (8.9 ± 1.4 mmol/l in the control, 9.0 ± 1.9 mmol/l in the obese untreated and 10.0 ± 2.1 mmol/l in the obese metformin-treated group, p=0.1 One-Way ANOVA). However, glucose tolerance at E17.5 (Fig. 2C) was impaired in the obese untreated dams compared to the controls (area under the curve (AUC): 526 ± 274 in obese untreated *vs.* 332 ± 108 in controls, *p*=0.02, n=20 and 20, insert in Fig. 2C). Metformin treatment reduced the AUC of the obese dams (AUC: 380 ± 189 in obese metformin-treated n=19), such that it was not different to the controls (p=0.7). Fasting insulin levels were increased in the obese untreated dams compared to controls (p=0.008, Fig. 2D), which was reduced with metformin treatment but still significantly increased compared to control dams (*p*=0.01, Fig. 2D). HOMA-IR was increased in the obese compared to controls (6.97 ±1.67 in control and 16.84 ±11.13 in obese untreated dams, p=0.01) and remained increased compared to the controls after metformin treatment (14.78 ± 9.22 in metformin-treated dams, p=0.03). In addition to impaired glucose tolerance and insulin resistance, obese untreated dams displayed increased liver fat compared to control dams (*p*<0.0001). This was reduced by metformin treatment (*p*=0.01 versus obese untreated group) but remained increased compared to controls (p <0.001), Fig.2*E* and *F*). Overall, metformin treatment in pregnancy resulted in improved metabolic health of the obese pregnant females.

### Metformin treatment in obese dams improves uterine artery compliance and reduces serum sFlt levels

Doppler ultrasound analysis of the uterine artery blood flow showed an increased pulsatility index (*p*=0.003) and increased resistance index (p=0.005) in the obese untreated dams compared to controls (Fig. 2*G, H*). The increased indices are indicative of high resistance in the vessel leading to impaired uterine artery blood flow. The increased uterine artery resistance in obese dams was rescued by metformin treatment. Both pulsatility and resistance index (*p*=0.04 and *p*=0.03 respectively) were significantly reduced in obese metformin-treated dams compared to obese untreated dams and no longer different to controls (p=0.5 and p=0.7 respectively, Fig. 2G, H). Maternal fasting insulin levels correlated positively with the uterine artery pulsatility index (Fig. 2I). Serum sFlt levels (soluble VEGFR-1) were increased significantly in the obese untreated group compared to control dams (37.3 ± 12.2 ng/mL in obese untreated *vs.* 26.2 ± 10.5 ng/mL in controls, *p*=0.04, n=13 and 14). Metformin treatment of obese dams reduced sFlt levels (29.5 ± 10.7 ng/mL, n=12) to levels that were not significantly different to controls (p=0.7).

### Fetuses from obese dams with and without metformin treatment are both symmetrically smaller than controls

**Figure 3:**
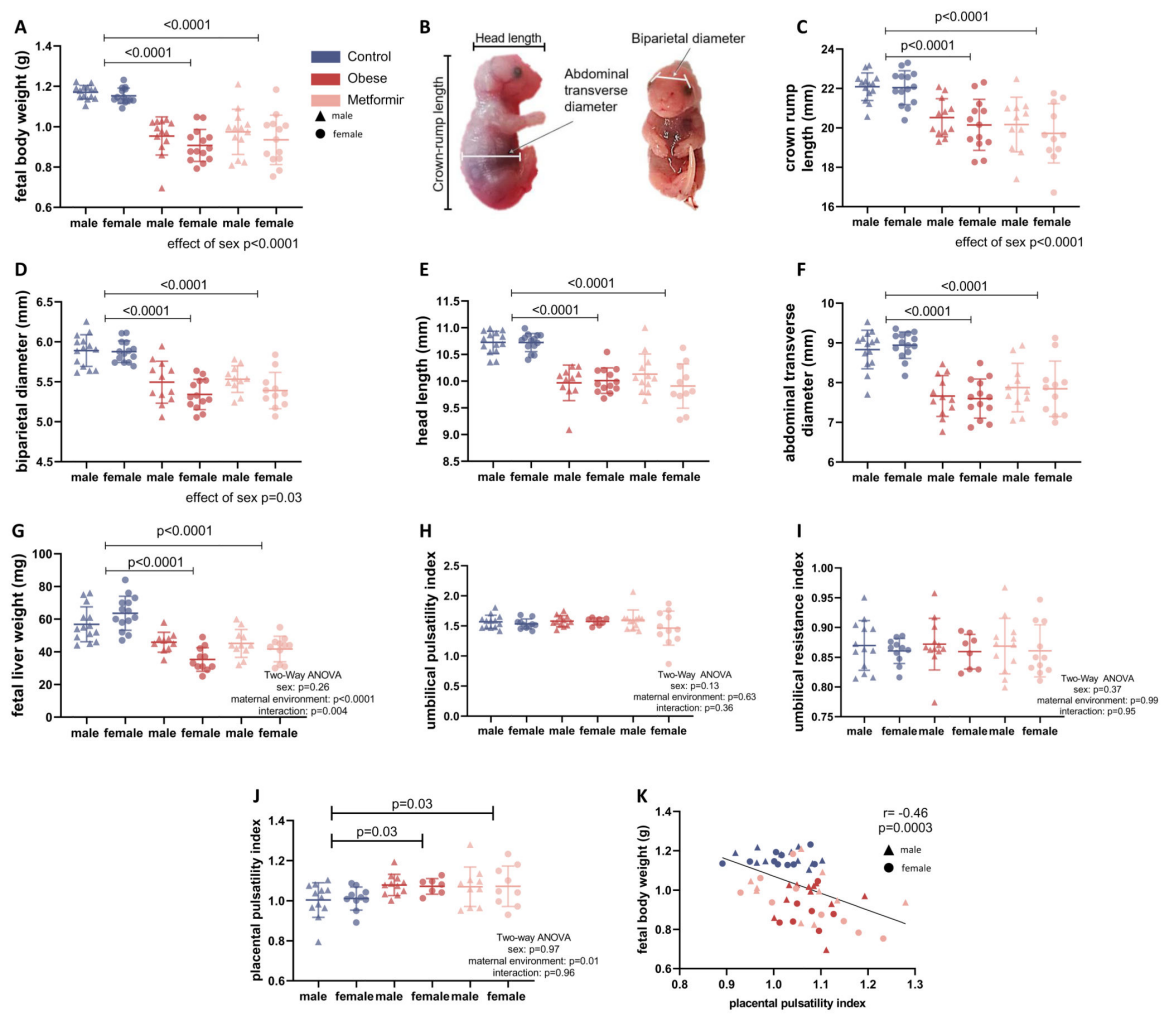
Fetuses from obese untreated and obese metformin-treated dams show symmetric growth restriction (A) Fetal weight was taken at E18.5 (n=14/14 dams for male/female control, n=13/14 dams male/female obese and n=13/13 dams for male/female metformin fetuses). (B) Fetal biometry was performed as shown and (C) crown-rump length, (D) biparietal diameter, (E) head length and (F) abdominal transverse diameter measured. For the fetal biometry n=14/14 dams for male/female control, n=12/13 dams for male/female obese and n=11/11 dams for male/female metformin fetuses are shown. Analysis is performed with a linear mixed model accounting for the dam as a random effect, in the graphs the mean ± SD per litter and sex is shown. (G) Fetal liver weights were taken at E18.5 in n=14/14 dams for male/female control, n=10/11 dams for female/male obese fetuses and n=11/11 dams, male/female metformin fetuses from mean ± SD and Two-Way ANOVA analysis is shown. (H) The umbilical pulsatility index (PI) and (I) resistance index were measured via ultrasound, analysis shows n=13/11 dams for male/female control, n=12/8 dams for male/female obese and n=12/11 dams for male/female metformin fetuses and Two-Way ANOVA (sex and maternal environment) with Tukey’s multiple comparison test. (J) The placental pulsatility index (PPI) was calculated via the following formula: mean uterine artery PI + mean umbilical artery PI) / 2, n=12/10 dams for male/female control, n=11/7 dams for male/female obese and n=10/9 dams for male/female metformin fetuses and Two-Way ANOVA analysis is shown. (K) The PPI was correlated with the fetal body weight, linear correlation and Pearson correlation coefficient r are shown.

Male and female fetuses from obese dams with and without metformin treatment weighed less compared to controls (*P*<0.001, Fig. 3*A*). Litter size was not significantly different between groups (7.9 ± 1.6 fetuses in the control, 8.4 ± 1.7 fetuses in the obese untreated and 8.6 ± 1.2 fetuses in the obese metformin-treated group, p=0.5 in One-Way ANOVA). The reduction in fetal weight was a result of symmetric growth restriction, with reductions of similar magnitudes in crown-rump-length, biparietal diameter, head length, abdominal transverse diameter and fetal liver weight (Fig. 3*B-G*). As expected, indices of growth were significantly lower in female fetuses compared to male fetuses (*P* <0.05). There were no significant differences in either the umbilical artery pulsatility index or resistance index (Fig. 3*H, I*), the middle cerebral artery pulsatility index, or the cerebroplacental ratio between any of the groups (data not shown). Placental pulsatility index (PPI), a measure for placental impedance and a tool to predict fetal growth restriction, was increased in the pregnancies of obese untreated and obese metformin-treated dams (Fig. 3*J*). The PPI was significantly correlated with fetal body weight (Fig. 3*K*).

### Placentas from obese untreated and obese metformin-treated dams have reduced labyrinthine area and increased calcification

**Figure 4:**
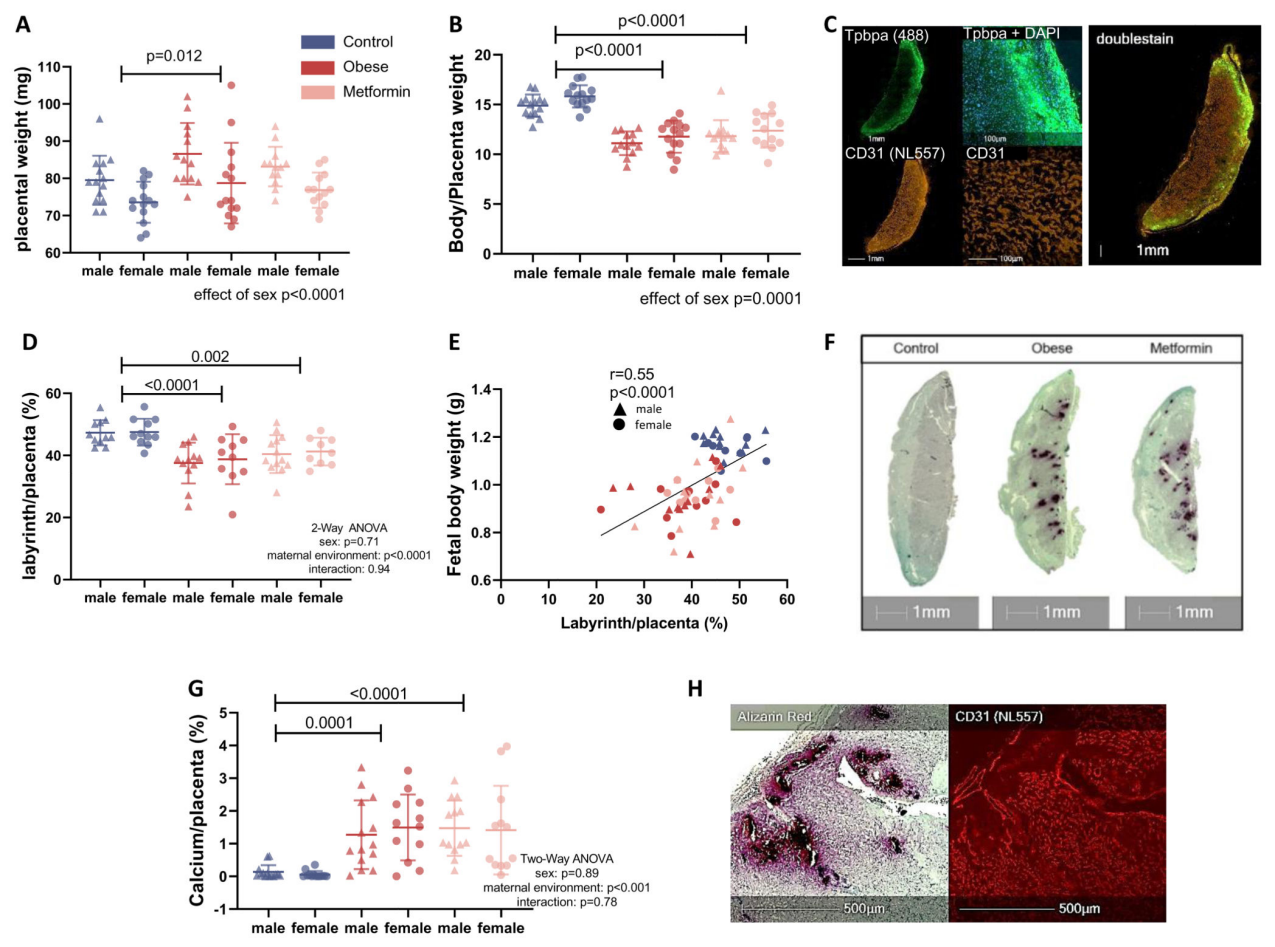
Placentas from obese untreated and obese metformin-treated animals show pathologies that can explain reduced efficiency (A) Placentas were weighed on the day of dissection (E18.5) and as an indicator of placental efficiency (B) the ratio of body to placental weight was calculated (n=14 dams for male and female control, n=13/14 dams for male/female obese and n=13 dams for male and female metformin placentas). (C) Placental sections were stained for the trophoblast cell marker Tpbpa and the endothelial cell marker CD31 via immunohistochemistry to allow delineation of the trophoblast and labyrinthine layer of the placenta (n=11 dams for male and female control, n=12/10 dams for male/female obese and n=13/9 dams for male/female metformin placentas). (D) The percentage of labyrinth to the whole placenta was then calculated. (E) Fetal body weight was correlated with the labyrinthine area of the placenta from that individual fetus, one male and one female fetus per litter was analysed, linear correlation and Pearson correlation coefficient r are shown. (F) The placentas were additionally stained for calcification with an Alizarin Red stain. (G) The areas stained with Alizarin Red are quantified and expressed as a percentage of the whole placenta (n=14 dams for male and female control, n=13/14 dams for male/female obese and n=13/12 dams for male/female metformin placentas). (H) In areas with calcium deposition the labyrinthine structure was damaged. Mean ± SD is shown and Two-Way ANOVA (sex and maternal environment) is performed with a Tukey’s multiple comparison test.

Male and female placentas from obese untreated but not obese metformin-treated dams were heavier compared to control placentas (*p*=0.007, Fig. 4A). The fetal bodyweight to placental weight ratio was reduced in both obese untreated and obese metformin-treated animals indicating reduced placental efficiency (*p*<0.001, Fig. 4B). Across all groups, placental efficiency was lower in male compared to female fetuses (*p*=0.001).

Male and female placentas from obese untreated dams had a reduced labyrinthine area (*p*<0.0001), the main nutrient exchange zone of the murine placenta (Fig. 4*C* and 4*D*). This reduction was not prevented by metformin treatment (*p*=0.002, Fig. 4*D*). Reduced placental labyrinth is likely to be a contributor to the reduced fetal growth as shown by the correlation between the labyrinthine area of the placenta and fetal weight (Fig 4*E*). Placental calcification was observed solely in the obese untreated and obese metformin-treated groups but not in the control group (*P*<0.001 for control *vs.* obese untreated and obese metformin-treated group, Fig. 4*F, G*). In areas with calcium deposits, the labyrinthine structure was damaged (Fig. 4*H*).

## Discussion

Exposure to a maternal high fat/high sugar diet resulted in a pronounced obesity phenotype and the subsequent development of glucose intolerance, insulin resistance, and reduced uterine artery compliance during pregnancy. Metformin treatment in our model resulted in improvement of maternal metabolic and vascular parameters but did not improve placental or fetal parameters.

Ultrasound imaging of the fetus and the uterine artery is commonly used to assess human pregnancies throughout gestation and a recent review highlighted the importance and new opportunities of pregnancy imaging in the field of developmental programming (Morrison *et al.,* 2021). The physiological drop in uterine artery PI via vascular remodelling during pregnancy is essential to enable low resistance placental blood flow and thus support fetal growth. In human pregnancy, maternal overweight/obesity is associated with an attenuation in the physiological drop in uterine artery PI (Teulings *et al.,* 2020) and there is increased likelihood of uterine artery PI above the normal range (Kim *et al.,* 2015). GDM has been associated with impairment of endothelium-dependent vasorelaxation (Knock *et al.,* 1997), which has been demonstrated in a murine GDM model with consequent increased uterine artery resistance (Stanley *et al.,* 2011). We show for the first time that our mouse model of maternal obesity recapitulates this, with increased levels of sFlt in the obese dams and reduced uterine artery compliance that correlates positively with maternal fasting insulin levels. Our obesity mouse model thereby recapitulates phenotypes of human obese pregnancies as obesity is a well-known risk factor for the development of preeclampsia (Roberts *et al.,* 2011). SFlt (VEGR-1), which is used as a biomarker for preeclampsia in humans, can bind vascular endothelial growth factor (VEGF) which leads to an angiogenic imbalance and endothelial dysfunction (Sones & Davisson, 2016). Metformin treatment in humans has previously been shown to reduce the incidence of preeclampsia and hypertensive disorders, potentially via increasing nitric oxide, improving endothelial dysfunction, and reducing sFlt secretion (Brownfoot *et al.,* 2016; Romero *et al.,* 2017; Soobryan *et al.,* 2018). In our model, metformin treatment improved uterine artery compliance and reduced sFlt in the maternal serum, adding novel evidence for metformin’s potential to prevent preeclampsia and demonstrating that our model provides an important platform to further elucidate mechanisms. Future work in this area will complement currently planned human trials of metformin for preeclampsia prevention (Cluver *et al.,* 2019).

Currently metformin is used to treat GDM in many settings, however there are wide global variations in clinical recommendations (Lindsay & Loeken, 2017). Long-term data about possible impacts of metformin use in pregnancy on offspring adiposity are starting to emerge, highlighting possible increased adiposity in mid-childhood following maternal metformin treatment (Tarry-Adkins *et al.,* 2019). Apart from teratology analyses that show no increased risk of fetal anomaly following maternal metformin exposure in pregnancy (Given *et al.,* 2018), little data exists from clinical studies regarding the direct impact of metformin on the placenta or fetus including growth (Tarry-Adkins *et al.,* 2019). The use of a mouse model allowed us to assess the effects of metformin exposure on the placenta and the fetus whilst controlling other factors (such as diet, housing conditions, genetic background). Sheep and rodent models are commonly used in the field of developmental programming significantly reducing the time to generate valuable data regarding safe and efficient interventions during pregnancy (Dickinson *et al.,* 2016). The murine pregnancy is well-characterised and thereby differences between the human and the murine pregnancy are well-known. The fetal period compared to the embryonic period is much longer in humans compared with the mouse that is born less mature. This is apparent when looking at the fat tissue development at birth with 1-2% of fat in a newborn mouse and 16% of fat in a newborn human (Widdowson, 1950). Although the human and the mouse both have chorioallantoic and hemochorial placentas, there are structural differences and the invasion of the placental trophoblast cells into the uterus is shallower in the mouse compared to the human (Schmidt *et al.,* 2015). However once the final placenta is established the labyrinthine zone in the mouse placenta and the chorionic villi in the human placenta are very similar with regards to the exchange mechanism between maternal and fetal blood (Rossant & Cross, 2001). The mouse is therefore a useful tool to address important questions in the field of developmental programming in relation to the placenta, such as those addressed in the current study.

In the current study, placentas from obese dams showed reduced placental efficiency, evidenced by increased calcium deposits, and reduced labyrinthine area. As the labyrinthine zone is the main exchange zone between the maternal and fetal circulation in the murine placenta the reduced size and the presence of calcium depositions is likely to restrict efficient nutrient transport to the fetus. Increased calcium depositions are associated with placental ageing in human pregnancy and are often observed in placentas from obese and GDM-affected pregnancies highlighting that our model mimics features of human pregnancies (Salge *et al.,* 2012). Mechanistic insight into how reduced labyrinthine area might occur comes from recent transcriptomic analyses from our laboratory showing downregulation of transcripts involved in labyrinthine development in placentas from obese dams (De Barros Mucci *et al.,* 2020). We observed a strong correlation between reduced labyrinthine area, increased placental impedance (as measured by the PPI (Gudmundsson *et al.,* 2017)), and reduced fetal growth. Many different factors on the maternal (such as suboptimal nutrition or smoking) and fetal side (such as genetic factors) can be associated with fetal growth restriction but a common feature and driving factor is reduced uterine-placental perfusion and reduced fetal nutrition (Nardozza *et al.,* 2017). It is thereby striking that despite the significant improvements in maternal metabolic health and uterine artery compliance observed with metformin treatment, the adverse impacts of maternal obesity on placental development were not rescued and fetal growth was still significantly restricted. We hypothesize that mechanisms driving the fetal growth restriction differ at least partially between the obese untreated and the obese metformin-treated pregnancies. Overall, the fetal weight and biometry data shows higher variation in the obese untreated and the obese metformin-treated group compared to the control group. This may reflect differential individual responses to the obesogenic diet and the metformin treatment. The maternal data highlights different degrees of obesity and glucose intolerance in our model that could contribute to the higher variability in these groups regarding fetal outcomes. In humans metformin treatment fails in 30-50% of women with GDM who then require additional insulin treatment (Tarry-Adkins *et al.,* 2020). A difference in the response to metformin treatment may therefore also explain increased variation in the metformin-treated group in our model.

We showed a strong correlation between metformin levels in the maternal and the fetal circulation and that circulating concentrations were equivalent. This result is consistent with human studies that demonstrate at least 50% of maternal metformin levels in fetal circulation (Priya & Kalra, 2018), with some studies showing equal or higher concentrations in the fetal circulation (Vanky *et al.,* 2005). Importantly, we demonstrate that as well as entering the fetal circulation, maternal administration of metformin also led to metformin uptake into fetal liver and kidney, both of which expressed high levels of known metformin transporters. Metformin was also present in the amniotic fluid, highlighting that the fetus is repeatedly exposed to metformin by swallowing. The immediate and long-term consequences of direct fetal tissue exposure to metformin are unknown. Data on metformin treatment outside of pregnancy shows that metformin activates AMPK and can inhibit complex I in mitochondria at high concentrations. Activation of AMPK leads to reduced mTOR signalling; this is relevant in highly-mitotic tissues such as cancer where metformin treatment can slow cell proliferation (Pernicova & Korbonits, 2014). Additionally, AMPK activation leads to reduced lipid synthesis and gluconeogenesis, mediating the beneficial effects on metabolic health in T2D patients (Rena *et al.,* 2017). Recent data shows that metformin increases GDF-15 which increases energy expenditure and reduces food intake and thereby reduces body weight (Coll *et al.,* 2020). It is possible that metformin has similar actions on fetal and placental tissues leading to altered metabolism and growth, that could have a negative impact, especially given the high degree of cell proliferation and division during development (Nguyen *et al.,* 2018). This could therefore contribute to the observed reduction in fetal growth despite the correction of uterine blood flow by metformin. It has been hypothesized that metformin may also have epigenetic effects on the fetus that could have long term health consequences via changes in activity of histone modification enzymes or DNA methylation (Bridgeman *et al.,* 2018; Owen *et al.,* 2021). This highlights the complexity of metformin use *in utero* and the need for further research in light of the lack of knowledge and understanding of metformin action in fetal tissues.

In conclusion, our study demonstrates that metformin has beneficial effects on maternal metabolic health and, consistent with human data, has the potential to prevent preeclampsia. However, despite the beneficial effects on maternal physiology, it did not prevent obesity-induced placental ageing and fetal growth restriction. Moreover, metformin enters the fetal circulation and highly proliferative fetal tissues, the long-term implications of which are currently unknown. These findings highlight the complex balance of risk versus benefits that are weighed in obstetric medical treatments and provide a well-characterised platform for further mechanistic research on pregnancies complicated by obesity and/or GDM and on the actions of metformin in pregnancy.

## Figures and Tables

**Table 1:** Taqman probes and their details

Gene symbol	Accession number	Assay ID	Amplicon length
Slc22a4	NM_019687.3	Mm00457739	73 bp
Slc47a1	NM_026183.5	Mm00840361	80 bp
Slc29a4	NM_146257.2	Mm00525575	82 bp
Slc22a3	NM_011395.2	Mm00488294	70 bp
Slc22a1	NM_009202.5	Mm00456303	69 bp
Slc47a2	NM_001033542.2	Mm02601013	64 bp
Slc19a3	NM_030556.2	Mm00472657	72 bp
Slc22a2	NM_013667.2	Mm00457295	85 bp
Slc22a5	NM_011396.3	Mm00441468	74 bp
Slc6a4	NM_010484.2	Mm00439391	72 bp
Slc6a2	NM_009209.3	Mm00436661	90 bp
Sdha (endogenous control)	NM_023281.1	Mm01352366	82 bp

## Data Availability

All analysed data can be found in the manuscript, raw datasets are available upon request.

## Key Points summary

- Maternal obesity and gestational diabetes mellitus have detrimental short- and long-term effects for mother and child.
- Metformin is commonly used to treat gestational diabetes mellitus in many populations worldwide but the effects on fetus and placenta are unknown.
- In a mouse model of diet-induced obesity and glucose intolerance in pregnancy we show reduced uterine artery compliance, placental structural changes and reduced fetal growth.
- Metformin treatment improved maternal metabolic health and uterine artery compliance but did not rescue obesity-induced changes in the fetus or placenta. Metformin crossed the placenta into the fetal circulation and entered fetal tissue.
- Metformin has beneficial effects on maternal health beyond glycaemic control. However, despite improvements in maternal physiology, metformin did not prevent fetal growth restriction or placental ageing. The high uptake of metformin into the placental and fetal circulation highlights the potential for direct immediate effects of metformin on the fetus with possible long-term consequences postnatally.
